# Generation of an artificial human B cell line test system using Transpo-mAb^TM^ technology to evaluate the therapeutic efficacy of novel antigen-specific fusion proteins

**DOI:** 10.1371/journal.pone.0180305

**Published:** 2017-07-13

**Authors:** Diana Klose, Mira Woitok, Judith Niesen, Roger R. Beerli, Ulf Grawunder, Rainer Fischer, Stefan Barth, Rolf Fendel, Thomas Nachreiner

**Affiliations:** 1 Fraunhofer Institute for Molecular Biology and Applied Ecology IME, Aachen, Germany; 2 Department of Experimental Medicine and Immunotherapy, Institute for Applied Medical Engineering, University Hospital RWTH Aachen, Aachen, Germany; 3 Institute of Molecular Biotechnology (Biology VII), RWTH Aachen University, Aachen, Germany; 4 NBE-Therapeutics AG, Basel, Switzerland; 5 South African Research Chair in Cancer Biotechnology, Institute of Infectious Disease and Molecular Medicine (IDM), Department of Integrative Biomedical Sciences, Faculty of Health Sciences, University of Cape Town, Cape Town, South Africa; Science and Technology Facilities Council, UNITED KINGDOM

## Abstract

The antigen-specific targeting of autoreactive B cells via their unique B cell receptors (BCRs) is a novel and promising alternative to the systemic suppression of humoral immunity. We generated and characterized cytolytic fusion proteins based on an existing immunotoxin comprising tetanus toxoid fragment C (TTC) as the targeting component and the modified *Pseudomonas aeruginosa* exotoxin A (ETA') as the cytotoxic component. The immunotoxin was reconfigured to replace ETA' with either the granzyme B mutant R201K or MAPTau as human effector domains. The novel cytolytic fusion proteins were characterized with a recombinant human lymphocytic cell line developed using Transpo-mAb™ technology. Genes encoding a chimeric TTC-reactive immunoglobulin G were successfully integrated into the genome of the precursor B cell line REH so that the cells could present TTC-reactive BCRs on their surface. These cells were used to investigate the specific cytotoxicity of GrB(R201K)-TTC and TTC-MAPTau, revealing that the serpin proteinase inhibitor 9-resistant granzyme B R201K mutant induced apoptosis specifically in the lymphocytic cell line. Our data confirm that antigen-based fusion proteins containing granzyme B (R201K) are suitable candidates for the depletion of autoreactive B cells.

## Introduction

B lymphocytes have both antibody-dependent and antibody-independent functions in the humoral immune system. In addition to the production of monoclonal antibodies, B cells release immunomodulatory cytokines and chemokines that influence the behavior of T cells and dendritic cells [1]. B cells are also responsible for antigen presentation, the regulation of lymphoid tissue organization, tissue regeneration, and wound healing. The specific function of peripheral B cells varies according to the B cell subset [1]. The dysregulation of B cell processing can contribute to the development of autoimmune diseases, e.g. aberrant receptor editing and deletions in several tolerance checkpoint genes increase the number of autoreactive B cell precursors [2]. Autoreactive B cells are hyperactive, and the secretion of autoreactive antibodies strongly influences the severity of pathogenesis [3–5]. Hyperactive autoreactive B cells also present autoantigens on the cell surface to stimulate pathogenic T cells. The abnormal recognition of autoantigens due to the breakdown of tolerance by autoreactive B and T cells leads to tissue damage [6, 7]. Systemic lupus erythematosus (SLE) is an autoimmune disorder characterized by an elevated autoantibody titer against nuclear proteins and/or DNA. An expanded subset of plasma blasts and plasma cells in the peripheral blood of patients with SLE is responsible for autoantibody secretion [8–10]. The treatment of autoimmune diseases such as SLE usually involves general immunosuppression and/or immunomodulation approaches that restore homeostasis, e.g. immunosuppressive agents such as the anti-malaria drug hydroxychloroquine, or immunomodulatory agents such as glucocorticoids, but these systemic treatments cause off-target effects that disrupt the immunological repertoire [5, 11–13].

Many standard therapeutic approaches for autoimmune diseases also affect healthy immune system cells, but research has focused recently on strategies for the specific elimination of pathogenic cell populations. Antibodies can be used for the targeted treatment of autoimmune diseases and there are four major mechanisms of action: ligand blocking, receptor blocking/modulation, downregulation of cell-surface receptor expression, and the depletion of antigen-presenting cells [14, 15]. Several human and chimeric antibodies have been developed that target receptors on the B cell surface such as CD19, CD20 and CD22, or B cell survival factors such as BAFF/BLyS and APRIL [13, 14, 16]. However, clinical studies have been mostly unsuccessful due to the failure to achieve clinical endpoints (safety and efficacy) or the prevalence of infection complications [17, 18]. The human monoclonal antibody belimumab, recognizing the B cell survival factor BLyS, is the only antibody that has been approved by the US Food and Drug Administration (FDA) for the treatment of SLE [17–20].

An alternative strategy to specifically eliminate autoreactive B cell populations involves the application of recombinant fusion proteins targeting B cells via their antigen-specific B cell receptors (BCRs). The fusion proteins consist of a cell-binding domain (an autoantigen or fragment thereof) fused to a toxin derived from plants or bacteria. This approach is equivalent to the use of immunotoxins, which were developed specifically to target malignant cell populations [21]. The cell-binding ligands in immunotoxins can be receptors, monoclonal antibodies or single chain variable fragments (scFvs). These are fused to a toxic domain such as the modified *Pseudomonas aeruginosa* exotoxin A (ETA'), only a few molecules of which are needed to inhibit protein synthesis and induce apoptosis [22]. Immunotoxins based on ETA' kill target cells efficiently, as demonstrated in several clinical trials [23–25]. In a previous study, we demonstrated that the antigen-specific targeting and depletion of a unique human B cell population was possible using an antigen-based ETA' fusion protein [26]. In this case, the cell-binding domain was an antigen fragment, the well-established tetanus toxoid fragment C (TTC), and the recombinant TTC-ETA' protein was tested for its ability to selectively bind and kill the murine TTC-reactive hybridoma cell line 5E4 as well as human tetanus-reactive memory B cells [26]. One drawback limiting the therapeutic impact of immunotoxins containing bacterial or plant toxins is their immunogenicity, particularly when repeated administration is necessary. This has been addressed by the development of a new generation of immunotoxins containing human cytolytic enzymes such as the serine protease granzyme B, the ribonuclease angiogenin, or the microtubule-associated protein tau (MAPTau) [21, 27–31]. We have developed mutated versions of these effector proteins to increase their potency, e.g. the granzyme B point mutant R201K is resistant to the natural granzyme B inhibitor serpin proteinase inhibitor 9 (PI-9), thus increasing its pro-apoptotic effect against target cancer cells [28, 30, 32]. Similarly, the mutated MAPTau protein includes two point mutations (S154K and S204K) at critical serine phosphorylation sites [33]. This non-phosphorylated MAPTau protein binds to microtubules, blocking the assembly and disassembly of the spindle microtubules, again enhancing its pro-apoptotic effects [34, 35]. Such enhanced human effector domains can also be also used to generate novel antigen-based fusion proteins for the antigen-specific elimination of B-cell populations via their unique BCRs.

The species-dependent reactivity of these human enzymes means that the murine TTC-reactive hybridoma cell line 5E4 cannot be used for the *in vitro* characterization of GrB(R201K)-TTC and TTC-MAPTau fusion proteins. Therefore, an artificial human cellular test system was required for the initial characterization of these proteins *in vitro*, including specific cell-binding, internalization and cytotoxicity [21, 36]. An alternative to human hybridoma cell lines is the generation of a specific TTC-reactive lymphocytic B cell line using a transposon-based vector system [37–39]. We used a system based on class II DNA cut-and-paste transposons, where the gene of interest is flanked by inverted terminal repeats (ITRs) that are recognized by the transposase [38, 40]. Transposon gene delivery systems have several advantages over viral vectors, including low cost, less innate immunogenicity and the potential to transfer larger DNA sequences [38, 40–42]. The *piggyBac* transposase is derived from the cabbage looper (*Trichoplusia ni*) and facilitates the efficient genetic modification of human cells [43–47]. The *piggyBac* transposase is included in the Transpo-mAb™ platform, which was developed as a mammalian cell display technology [39]. This allows the integration of human immunoglobulin genes into mammalian host cells, resulting in antibody presentation or expression in a soluble form. The transposase system consists of one plasmid encoding the *piggyBac* transposase and two further vectors, one each for the human antibody heavy chain and light chain variable genes (V_H_ and V_L_), plus in each case an internal ribosomal entry site (IRES) and a selectable marker such as an antibiotic resistance gene or the enhanced green fluorescent protein (eGFP), all flanked by the ITRs [39]. These Transpo-mAb^TM^ vectors were used to generate a stable human lymphocytic cell line presenting a TTC-specific antibody on the surface, i.e. a TTC-specific BCR. The variable chain sequences of the TTC-reactive murine hybridoma antibody 5E4 [48] were transferred into the vectors containing the ITRs. By co-transfection with an expression vector carrying the *piggyBac* transposase gene (hy-PB), the transposase was transiently expressed resulting in the insertion of the TTC-antibody V_L_ and V_H_ sequences randomly into the genome of REH host cells, a human precursor B cell line derived from an acute lymphocytic leukemia patient [49].

Here we describe the generation of a novel transgenic human lymphocytic cell line specific for TTC. The engineered human B cell line was used to demonstrate the receptor-mediated specific cell binding and internalization of TTC-based fusion proteins. We were thus able to demonstrate the specific toxicity of novel antigen-based fusion proteins with human effector domains.

## Materials and methods

### Cell lines and cell culture

TTC-reactive 5E4 hybridoma cells (kindly provided by Prof. Dr. M. Shapiro, Rockville, USA) and REH cells were cultured under standard conditions (RPMI 1640 medium + GlutaMax™ including 10% fetal calf serum (FCS), 100 U/ml penicillin, 100 mg/ml streptomycin, at 37°C, 5% CO_2_). Transfected TTC-reactive REH cells (ATCC® CRL-8286™) were cultured after sorting in RPMI 1640 medium + GlutaMax™, 20% FCS, 100 U/ml penicillin, 100 mg/ml streptomycin, at 37°C, 5% CO_2_ for 1 week to stimulate cell proliferation. Afterwards, the transfected REH cells were cultured under standard conditions.

### RNA isolation and cDNA synthesis

RNA was isolated from 5E4 hybridoma cells using the M&N NucleoSpin RNA II Kit (Macherey-Nagel, Düren, Germany) according to the manufacturer’s instructions, and the RNA quality was verified by agarose gel electrophoresis. First-strand cDNA was synthesized using the First Strand cDNA Synthesis Kit (Thermo Fisher Scientific, Schwerte, Germany) and the oligo(dT) primers provided in the kit with 1 μg of RNA, according to manufacturer’s instructions.

### V-gene DNA amplification

The V_H_ and V_L_ sequences of the TTC-reactive antibody from the murine hybridoma cell line 5E4 were identified by V-gene rescue polymerase chain reaction (PCR) with the primer set described in our earlier report [50]. The V_L_ and V_H_ sequences were amplified using Phusion high-fidelity DNA polymerase (Thermo Fisher Scientific), 200 nM of each primer and 1 μl of cDNA in a 50-μl reaction volume. After heating the reaction to 98°C for 30 s, we carried out 28 amplification cycles at 98°C for 8 s, 57°C for 10 s, and 72°C for 15 s, then a final extension step at 72°C for 5 min, in a Veriti 96-well thermocycler (Applied Biosystems, Thermo Fisher Scientific). The PCR products were separated by 1.2% (w/v) agarose gel electrophoresis and purified using the M&N Nucleo Extraction Kit II (Macherey-Nagel) according to the manufacturer’s instructions.

### Construction of transposase-based vectors

**Fig 1:**
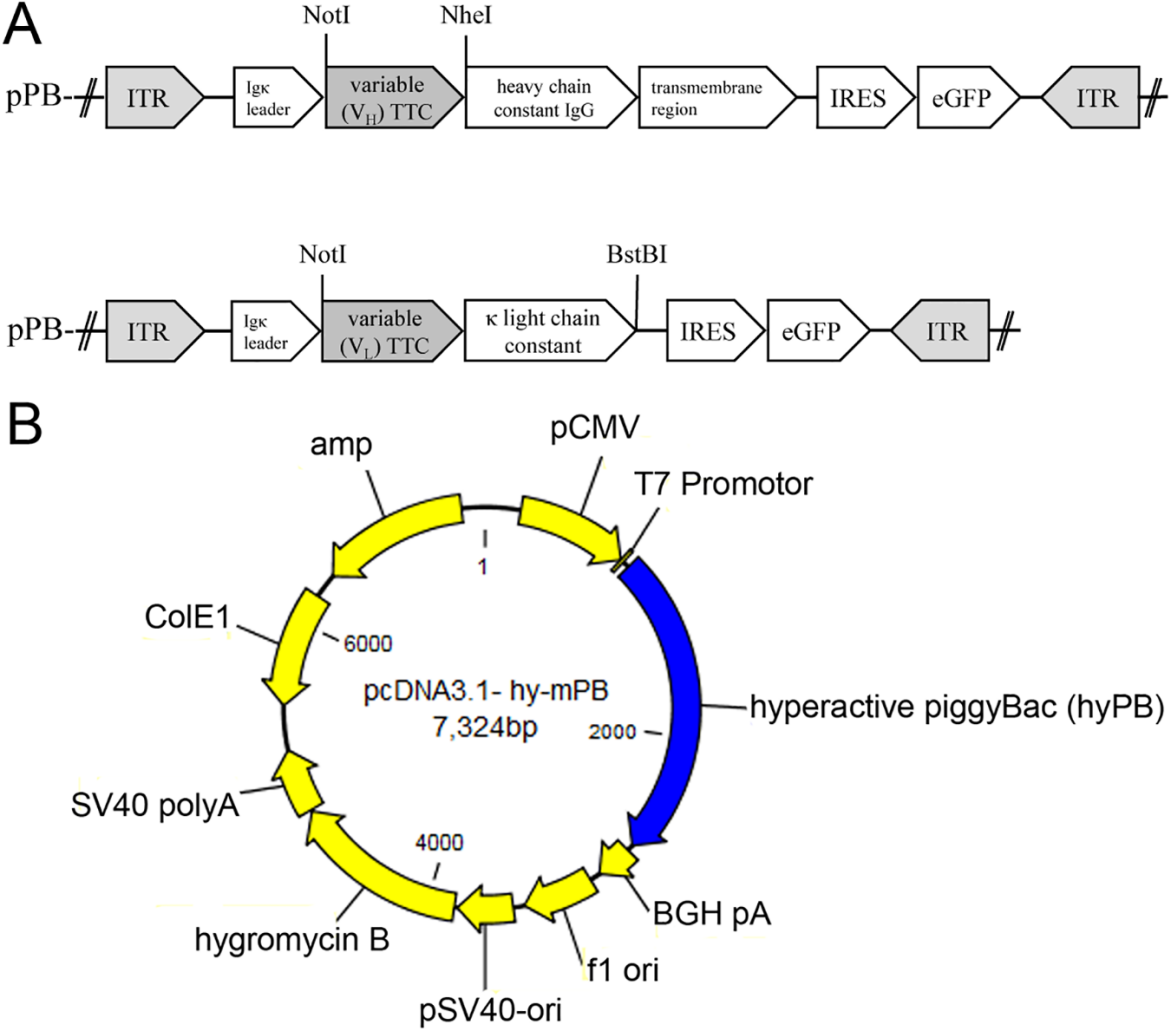
The transposon-based eukaryotic expression vectors. (A) The eukaryotic expression vectors pPB-EGFP-V_H_-TTC and pPB-EGFP-V_L_-TTC. The variable chain fragments for the TTC-specific antibody were transferred to the eukaryotic expression vector pPB-EGFP using the NotI and NheI or BstBI restriction sites. Abbreviations: Ig kappa = murine signal sequence for protein secretion into the cell culture supernatant, IRES = internal ribosome entry site for the co-expression of eGFP, eGFP = enhanced green fluorescent protein, ITR = inverted terminal repeats for transposase recognition. (B) Transposase expression vector pcDNA3.1-hy-mPB, expressing the mammalian-optimized hyperactive piggyBac transposase. Abbreviations: pCMV = cytomegalovirus promoter, T7 promoter = IPTG-inducible promoter + *lac* operator, BGH pA = bovine growth hormone polyadenylation signal, hygromycin B = marker gene for the selection of transfected cells, f1 ori = origin of replication for production of single-stranded DNA by M13 helper phage, pSV40 = early SV40 promoter, SV40 polyA = polyadenylation signal, ColE1 origin = bacterial origin of replication, amp = ampicillin resistance gene for the selection of transformed *E*. *coli*.

The V_L_ and V_H_ sequences of the TTC-reactive antibody clone 5E4 were transferred to vector pJET1.2 using the CloneJET PCR Cloning Kit (Thermo Fisher Scientific) according to the manufacturer’s instructions. After the ligation step, the plasmids were introduced into *Escherichia coli* strain DH5α by heat shock and plasmid DNA was prepared using the M&N NucleoSpin Kit (Machery Nagel). After PCR amplification of the antibody fragments using the primers listed in Table 1, the sequences were transferred to vector pTT5 [51] by ligation at the AgeI/BsiWI (V_L_ fragment) or AgeI/SalI (V_H_ fragment) sites [52]. The V_L_/V_H_ fragments from vectors pTT5-TTC-V_L_/V_H_ were then amplified, including the Ig-kappa leader sequence from the pTT5 vector, and transferred to the pPB-EGFP transposase-based vectors [37, 39] using the restriction sites NotI/BstBI (V_L_ fragment) or NotI/NheI (V_H_ fragment) in order to generate the final vector pPB-V_L_/V_H_-TTC-EGFP (Fig 1A). Sequencing was carried out on an ABI Prism^®^ 3730 Genetic Analyzer (Applied Biosystems).

**Table 1:** Oligonucleotides for PCR amplification.

Primer	Sequence
AgeI-VL-TTC-fwd	CTTACTAACCGGTGTACATTCTGACATCCAGATGACTCAG
BsiWI-VL-TTC-rev	TAGTAAGCGTACGTTTGATTTCCAGCTTGGTGCC
AgeI-VH-TTC-fwd:	CTTACTAAACCGGTGTGCACTCCGAGGTGCAGCTGAAGGAGTC
VH-TTC-Sal-rev	TAGTAAGGTCGACGCTGAGGAGACGGTGACTGAGG
BstBI-LCpTT5-rev	TAGTAAGTTCGAACTCTAGACTAACACTCTCCCCTGTTGAAG
VH-TTC-NheI-rev	CTTACTAGCGGCCGCCATGGGATGGTCATGTATCATCC
NotI-leader-fwd	CTTACTAGCGGCCGCCATGGGATGGTCATGTATCATCC

### Transfection of REH cells

To generate a human TTC-specific cell line, REH cells were co-transfected with the plasmids pcDNA3.1-hy-mPB and pPB-V_L_/V_H_-TTC-EGFP. Prior to transfection, 30 μg of the vectors pPB-V_L_-TTC-EGFP and pPB-V_H_-TTC-EGFP, plus 10 μg of pcDNA3.1-hy-mPB, was prepared in 400 μl RPMI 1640 medium without supplements. We then washed 5 x 10^6^ REH cells twice with RPMI 1640 medium without supplements and re-suspended them in 3 ml of the same (pre-warmed) medium. The plasmid DNA mixture was added to 400 μl of cell suspension (1.7 x 10^6^ cells/ml) and transferred into an electroporation cuvette (BioRad, Munich, Germany). Electroporation was carried out using the GenePulser System (BioRad) at 250 V and 950 μF. After 10 min incubation at room temperature, the cells were washed, re-suspended (RPMI 1640 medium + GlutaMax™, 10% FCS, 100 U/ml penicillin, 100 mg/ml streptomycin) and cultivated at 37°C and 5% CO_2_. REH cells were co-transfected in parallel as described above with the pPB-EGFP-LC-Ac10/pPB-EGFP-HC-Ac10 transposon-based expression vectors described earlier [37] to generate a control cell line (mock-reactive REH cells).

### Fluorescence activated cell sorting and monitoring of transfected REH cells

Transfected TTC-reactive REH cells were sorted by fluorescence-activated cell sorting (FACS) using the BD Influx cell sorter (BD Bioscience, Franklin Lakes, New Jersey). Positive transfected REH cells express the reporter protein eGFP and the TTC-specific antibody on the cell surface. The antibody-presenting REH cells were distinguished using a TTC-based fusion protein (SNAP-TTC-BG-647) covalently coupled to a benzylguanine modified fluorescent dye [26]. The fluorescence signals of the eGFP reporter protein and the bound SNAP-TTC-BG-647 protein were used to isolate double-positive TTC-reactive REH cells from the cell population. In the case of the mock-transfected REH cells, FACS was carried out using the eGFP signal and the anti-human-Ig-kappa antibody conjugated to PE-Cy7. Double-positive REH cells were cultivated in RPMI 1640 medium (+ GlutaMax™, 20% FCS, 100 U/ml penicillin, 100 mg/ml streptomycin) at 37°C and 5% CO_2_. To monitor the sorted TTC-reactive REH cells, FACS was carried out using the SNAP-TTC-BG-647 protein [26]. Cultured TTC-reactive or mock-transfected REH cells were collected, washed in FACS staining buffer (1x PBS, 5 mM EDTA, 2% v/v FCS) and incubated with 1 μg SNAP-TTC-BG-647 protein or anti-human-Igkappa-PE-Cy7 antibody (diluted 1:200 in FACS staining buffer) for 20 min on ice in the dark. After washing with FACS staining buffer, the fluorescence signal from the bound protein was measured using a FACSVerse™ device (BD Bioscience) to analyze the presentation of BCRs on the cell surface.

### Cloning of eukaryotic expression vectors and protein expression

Cytolytic fusion proteins containing granzyme B variant R201K or MAPTau were expressed in HEK 293T cells. The synthetic TTC DNA sequence (GenBank accession no. FJ917402.1) prepared by GeneArt^®^ Gene Synthesis (Thermo Fisher Scientific) was flanked with NotI/SfiI sites. This synthetic TTC DNA sequence was inserted at the same sites in vector pMS [53] to yield final expression plasmids pMS-EGrB(R201K)-TTC and pMS-TTC-MAPTau. The integrity of the vectors was confirmed by DNA sequencing. Human embryonic kidney cells (HEK293T, ATCC, Wesel, Germany, CRL-11268) were cultured under standard conditions RPMI 1640 medium, 10% v/v fetal calf serum (FCS), 100 U/ml penicillin, 100 mg/ml streptomycin, 37°C, 5% CO2). 3x10^5^ HEK 293 T cells per well in a 6-well plate were transfected with the expression vectors (pMS-EGrB(R201K)-TTC and pMS-TTC-MAPTau) using Roti^®^Fect (Carl Roth) according to the manufacturer’s instructions. Transfected cells were cultured with 100 ng/ml Zeocin® (Invitrogen, Carlsbad, USA) for selection. The supernatant was collected from the transfected HEK293T cells and the proteins were purified by immobilized metal-ion affinity chromatography (IMAC).

### Protein purification

Supernatants were collected from transfected HEK 293T cells so that the cytolytic fusion proteins EGrB(R201K)-TTC and TTC-MAPTau, and the mock-proteins EGrB(R201K)-H22 [54] and IL3-MAPTau (kindly provided by Christoph Stein) was purified by immobilized metal-ion affinity chromatography (IMAC) using a nickel-Sepharose (Ni-NTA) Superflow Cartridge (Qiagen, Hilden, Germany) on a ÄKTApurifier system (GE Healthcare, Chicago, Illinois) as previously described [30, 54–56]. The EGrB(R201K)-TTC protein was dialyzed against 20 mM Tris-HCl, 200 mM NaCl (pH 7.4) and the other proteins were dialyzed against PBS using a 6000–8000 Da molecular weight cutoff (MWCO) ZelluTrans dialysis membrane (Car Roth, Karlsruhe, Germany) and all the proteins were concentrated using 30,000 Da MWCO Vivaspin columns (GE Healthcare). Purified proteins were separated by SDS-PAGE and visualized by Coomassie Brilliant Blue staining against Color Prestained Protein Standards, Broad Range (New England Biolabs, Ipswich, Massachusetts). Western blot analysis was carried out using an anti-polyhistidine antibody (Thermo Fisher Scientific) diluted 1:5000 in PBS, and a secondary goat anti-mouse IgG (Fc-specific) antibody (Sigma-Aldrich, Munich, Germany) conjugated to alkaline phosphatase (diluted 1:5000 in PBS). The protein concentration in the samples was determined using an AIDA Analyzer (Raytest GmbH, Straubenhardt, Germany) with bovine serum albumin (BSA) as the standard.

### Internalization assay

Internalization was investigated using the SNAP-TTC-BG-647 protein [26]. TTC-specific REH cells and the control cells were washed with staining buffer (PBS, 2% FCS, 5 mM EDTA) and re-suspended in RPMI 1640 medium + GlutaMax™, 10% FCS, 100 U/ml penicillin and 100 mg/ml streptomycin, including 1 μg SNAP-TTC-BG-647 protein per sample. Per sample, 1 x 10^5^ cells were incubated with the protein at 37°C or 4°C for 30–90 min in the dark. Afterwards, the cells were washed with 2 ml PBS to remove unbound protein and then incubated with Hoechst 33342 (4 μg/μl final concentration) for nuclear staining. The stained cells were transferred directly to a microscope slide and visualized by confocal microscopy using a Leica TCS SP8, LAS AF (Leica Microsystems, Wetzlar, Germany). Images were processed using ImageJ 1.50f software (Wayne Rasband, National Institutes of Health, Bethesda, Maryland).

### Enterokinase digest

The fusion protein EGrB(R201K)-TTC was activated with one unit of recombinant enterokinase (Merck Millipore, Darmstadt, Germany) per 50 μg protein in 2 mM CaCl_2_ in Tris-HCl buffer (20 mM Tris-HCl, 200 mM NaCl, pH 7.4) and then incubated for 16 h at 23°C. The digested GrB(R201K)-TTC protein was exchanged into PBS using a MiniTrap™ G25 desalting column (GE Healthcare) and the activated protein was stored at –20°C.

### Granzyme B substrate assay

The enzymatic activity of the GrB(R201K)-TTC fusion protein was determined using a granzyme B substrate assay with 500 ng of the synthetic substrate Ac-IETD-*p*NA (Calbiochem/Merck, Darmstadt, Germany). Enzyme kinetics was measured for 60 min at 2-min intervals in 96-well plates on an Epoch Microplate Spectrophotometer (BioTek, Bad Friedrichshall, Germany). All measurements were taken in duplicate.

### Binding of recombinant proteins to TTC-reactive human lymphocytic REH cells

We washed 1–5 x 10^5^ REH cells (TTC^+^ or control) with FACS staining buffer and incubated them with 100 nM of each recombinant protein for 20 min on ice. After washing in FACS staining buffer the cells were pelleted (500 x g, 5 min, 4°C) and incubated with an anti-polyhistidine antibody conjugated with phycoerythrin (PE) (clone GG11-8F3.5.1, Miltenyi, Bergisch Gladbach, Germany) for 20 min on ice in the dark. After a final washing step, the signal was measured on the FACSVerse™ instrument and analyzed using FACSuite software v1.05.

### Cell viability and apoptosis assays

An XTT-based cell viability assay was used to determine the toxicity of the TTC-based proteins against the hybridoma cell line 5E4 and human lymphocytic TTC-reactive REH cells. The cells (1x10^4^ cells per well) were incubated with serial dilutions of each recombinant protein for 72 h at 37°C and 5% CO_2_ in a 96-well plate. We also set up controls of untreated cells (100% proliferation) and cells treated with zeocin (0% proliferation). We added 50 μl XTT/phenazine methosulfate and incubated the cells for 4 h at 37°C and 5% CO_2_ before measuring the absorbance at 450 nm and the reference wavelength at 630 nm in an Epoch Microplate Spectrophotometer. The half maximal inhibitory concentration (EC_50_) of each protein was estimated using the three-parameter dose-response curve fit equation and the significance of the dose-response effect was estimated by two-way analysis of variance (ANOVA) with Bonferroni’s post hoc test using GraphPad Prism v5 software (GraphPad Software Inc., San Diego, California).

Annexin V/propidium iodide (PI) staining was used to determine the pro-apoptotic effect of the recombinant proteins TTC-ETA', GrB(R201K)-TTC and TTC-MAPTau. TTC-reactive REH cells and control cells (1x10^5^ cell per sample) were incubated with 50 nM of each recombinant protein or with camptothecin as a positive control in a 24-well plate for 72 h at 37°C and 5% CO_2_. The cells and the supernatant were then transferred to a FACS tube and washed with 500 μl annexin buffer (10 nM HEPES, 140 mM NaCl, 25 mM CaCl_2_, pH 7.4). The cells were then pelleted (500 x g, 5 min, 4°C) and incubated with annexin V allophycocyanin (APC) conjugate diluted 1:50 (eBioscience, San Diego, California) for 20 min in the dark. After a final washing step with annexin buffer, the cells were re-suspended in annexin buffer and stained with 1 μg/ml PI before analysis in a FACSVerse instrument. The different states of the cells (viable, early apoptotic and late apoptotic/necrotic) were determined and analyzed by one-way ANOVA followed by the Bonferroni’s post hoc test using GraphPad Prism v5.

## Results

### Generation of a TTC-reactive lymphocytic REH cell line using the piggyBac transposon system

Human lymphocytic REH cells presenting TTC-reactive antibodies were prepared by isolating the DNA sequences encoding the TTC-specific antibody produced by the murine hybridoma cell line 5E4. The murine hybridoma cells were harvested, RNA was isolated and reverse transcribed into cDNA. The specific V_H_ and V_L_ fragments were amplified by PCR and integrated into expression vectors containing the transposon sequences (Fig 1A). The transposase system requires co-transfection with a plasmid providing the transposase. The lymphocytic REH cells were therefore simultaneously co-transfected with the V_H_/V_L_ transposon plasmids (Fig 1A) and pcDNA3.1-hy-mPB containing the *piggyBac* transposase gene (Fig 1B).

**Fig 2:**
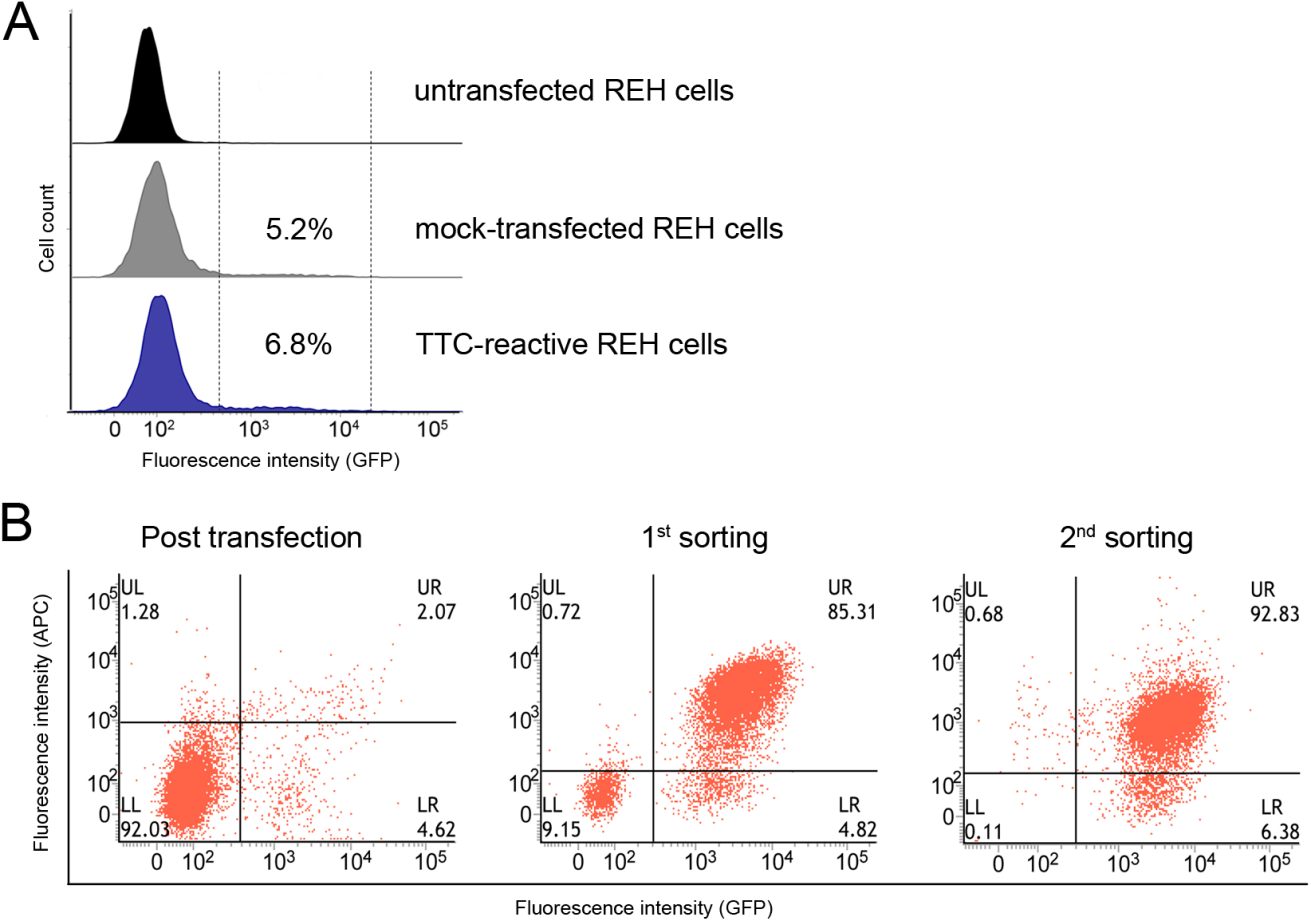
Analysis of transfection efficiency and the enrichment of TTC-reactive REH cells by cell sorting. (A) The transfection efficiency of transfected REH cells was analyzed by FACS. Using the fluorescence signal of the reporter protein eGFP. Untransfected REH cells showed no eGFP fluorescence signal (top) compared to the transfected REH cells, TTC-reactive REH cells (middle) and the mock-transfected REH cells (below). (B) FACS analysis for TTC-reactive BCRs on the surface of transfected REH cells after two sorting rounds. TTC-reactive BCRs on the human REH cells were identified by staining with 100 nM SNAP-TTC-BG-647. FACS analysis detected the fluorescence signals from bound SNAP-TTC-BG-647 protein and the reporter protein eGFP. After the second round of sorting, the proportion of double positive (APC^+^ / FITC^+^) TTC-reactive REH cells was estimated.

FACS analysis for the reporter protein eGFP indicated a transfection efficiency of ~7% for both transfection steps (TTC-reactive, mock-reactive plasmids) compared to untransfected REH cells (Fig 2A). Positive TTC-reactive REH cells carrying the specific antibody on the cell surface were analyzed using the SNAP-TTC-BG-647 protein. FACS analysis revealed the specific binding of SNAP-TTC-BG-647 to the TTC-reactive cell population (2% of the REH cells presented the TTC-reactive BCR on the cell surface). The population of transfected REH cells that achieved strong binding to the SNAP-TTC-BG-647 protein, indicated by a positive fluorescence signal in the APC channel, was sorted and cultivated (Fig 2B). The TTC-reactive REH cell population was 85% enriched for BCR-positive cells after the first sorting round (Fig 2B, middle) and this increased to more than 92% after the second round (Fig 2B, right). A negative control cell line was generated in the same way using the pPB-LC/HC-AC10-EGFP expression vectors [39], encoding the CD30-specific monoclonal antibody AC10 (mock-transfected REH cells) (Fig 2A). These control cells were also sorted and cultivated as above and were used as negative controls in subsequent experiments.

### Internalization of the fusion proteins by the TTC-reactive lymphocytic REH cell population

**Fig 3:**
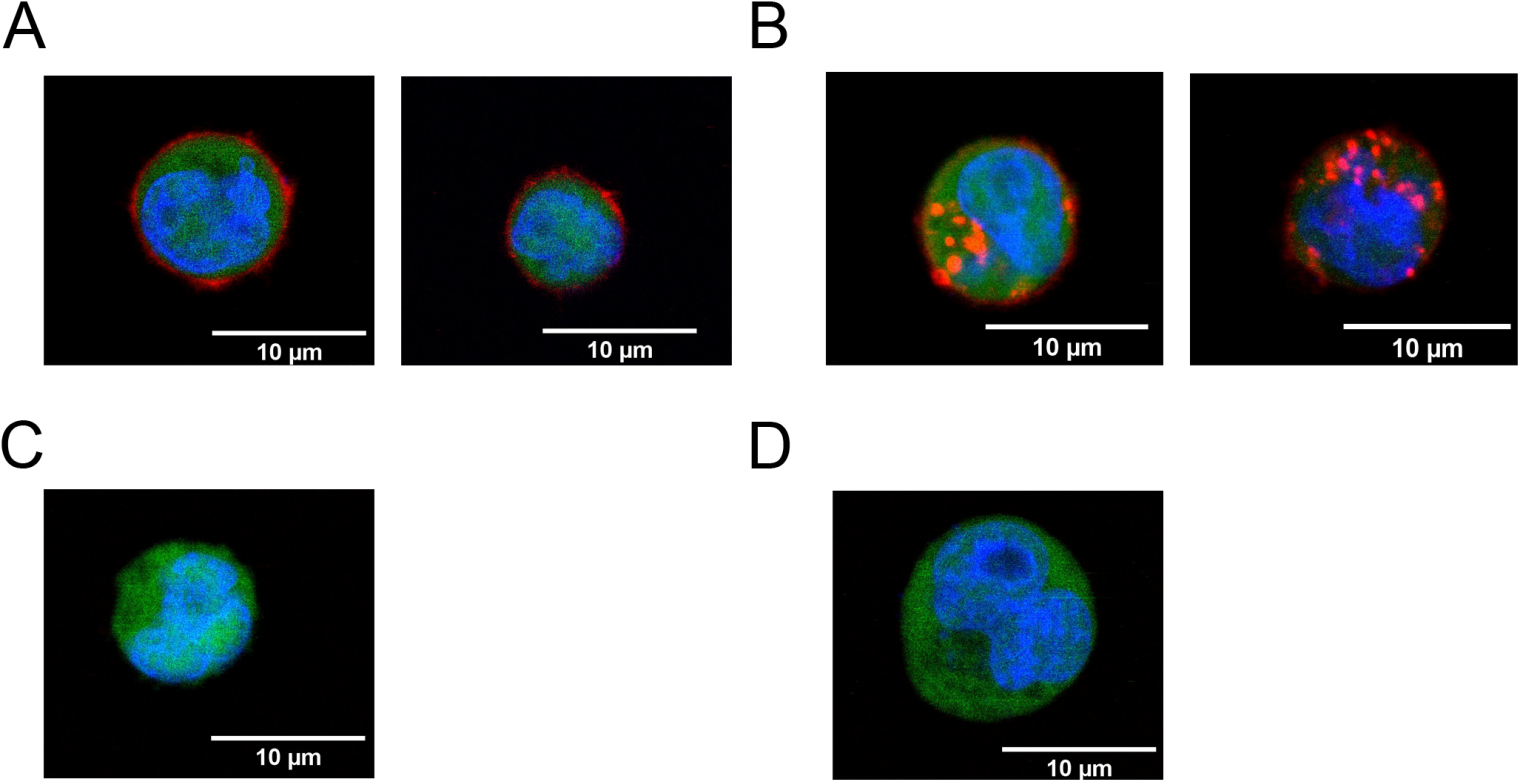
Internalization of SNAP-TTC-BG-647 by TTC-reactive REH cells. The TTC-reactive REH cells were incubated with SNAP-TTC-BG-647 at 4°C (**A**) or 37°C (**B**) and the cells were observed by confocal microscopy. As negative controls, TTC-reactive REH cells were incubated without protein (**C**) and mock-transfected REH cells were incubated with SNAP-TTC-BG-647 for 30 min at 37°C (**D**). Images of the eGFP (excitation = 488 nm), SNAP-TTC-BG-647 (excitation = 647 nm) and nuclear counterstaining (excitation = 405 nm) signals were merged using ImageJ v1.50f software. Scale bar = 10 μm.

Internalization assays were carried out to characterize the functionality of the novel fusion proteins in the presence of human TTC-reactive REH cells. When the cells were incubated at 4°C, specific binding of the SNAP-TTC-BG-647 protein was observed (Fig 3A). Efficient TTC-specific internalization of the protein was demonstrated using SNAP-TTC-BG-647, revealing the intracellular accumulation of the red-labeled protein after incubation at 37°C for 30 min (Fig 3B) compared to the untreated TTC-reactive REH cells (Fig 3C). The control mock-transfected REH cells showed no evidence of nonspecific protein binding and internalization (Fig 3D).

### Generation and characterization of the GrB(R201K)-TTC cytolytic fusion protein

**Fig 4:**
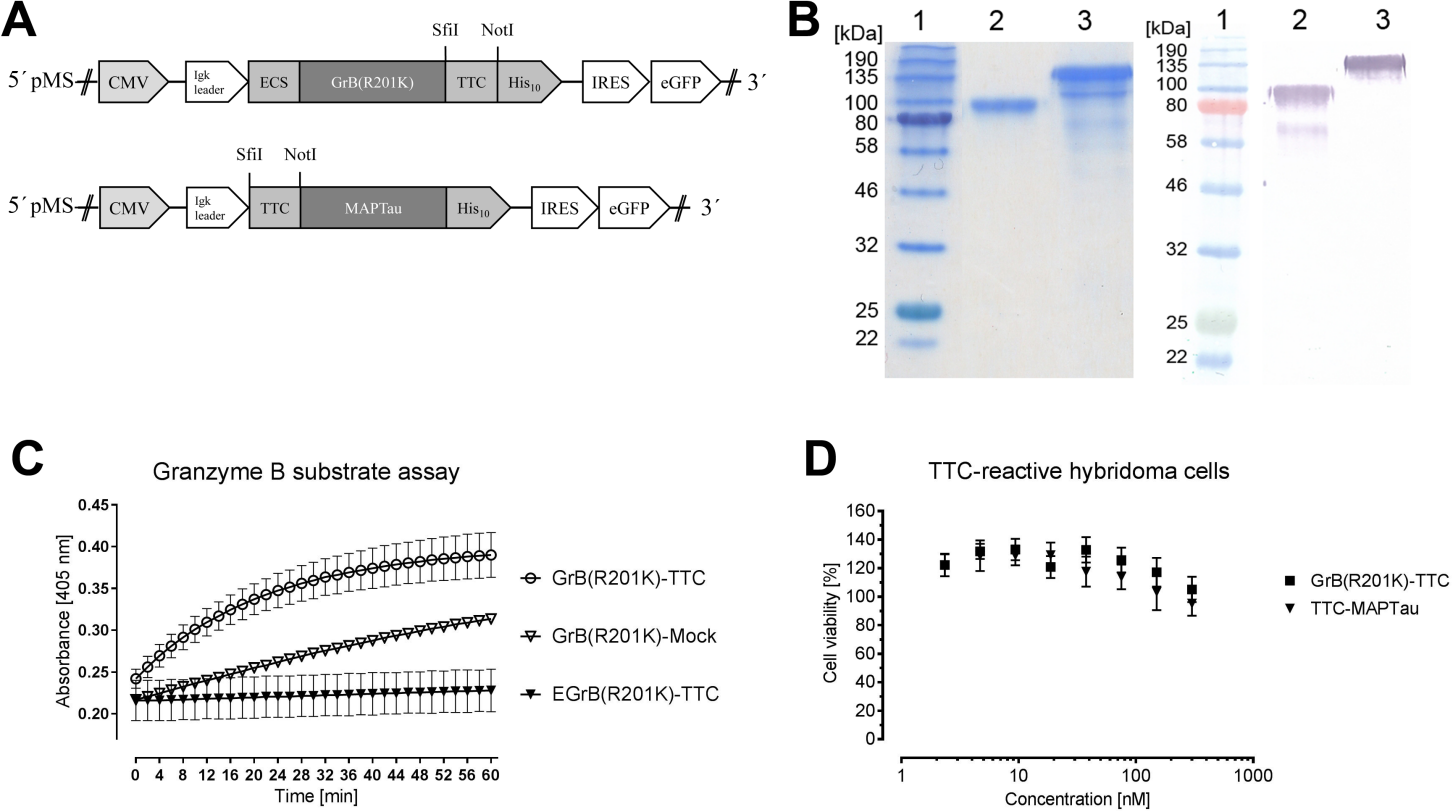
Expression and characterization of TTC-based fusions proteins. (A) The TTC DNA sequence was transferred to the pMS vector system using the SfiI/NotI sites to generate pMS-EGrB(R201K)-TTC and pMS-TTC-MAPTau. Abbreviations: CMV = cytomegalovirus promoter, Ig kappa = murine signal sequence for protein secretion into the cell culture supernatant, ECS = enterokinase cleavage site, His_10_ = polyhistidine tag, IRES = internal ribosome entry site for the co-expression of eGFP, eGFP = enhanced green fluorescent protein. (B) EGrB(R201K)-TTC and TTC-MAPTau were expressed in HEK 293T cells and purified by IMAC. The GrB-(R210K)-TTC and TTC-MAPTau proteins were separated by denaturing SDS-PAGE followed by staining with Coomassie Brilliant Blue (left). Western blot analysis (right) using anti-polyhistidine and goat anti-mouse IgG (Fc specific) antibodies revealed protein bands of the anticipated sizes for GrBR201K-TTC (80 kDa) and TTC-MAPTau (93 kDa). Lane 1—Color Prestained Protein Standard, Broad Range (11–245 k); lane 2—GrBR201K-TTC, lane 3—TTC-MAPTau. (C) EGrB(R201K)-TTC was digested with enterokinase and granzyme B activity was tested using substrate Ac-IETD-*p*NA (white circle) compared to uncleaved EGrB(R201K)-TTC (black triangle) and the mock-protein (white triangle). The enzymatic activity of the granzyme B domain was determined using a colorimetric assay and the absorbance at 405 nm was monitored for 60 min in 2-min intervals. (D) An XTT-based cell viability assay was carried out using serial dilutions of the novel TTC-fusion proteins against the mouse TTC-reactive hybridoma cell line 5E4 (72 h, 37°C, 5% CO_2_). The data are means ± standard deviation (SD) of technical triplicates of three independent experiments (n = 3).

The TTC DNA sequence was transferred to pMS expression vectors containing the coding sequences for cytotoxic domains GrB(R201K) and MAPTau (Fig 4A) and the recombinant vectors were verified by DNA sequencing. HEK 293T cells were transfected with each of the vectors and the recombinant fusion proteins EGrB(R201K)-TTC and TTC-MAPTau were purified from the cell supernatant by IMAC with yields of 4.5 mg/l EGrB(R21K)-TTC and 1.6 mg/l TTC-MAPTau (Fig 4B). EGrB(R201K)-TTC was activated by *in vitro* enterokinase digestion to expose the N-terminal enterokinase site and the proteolytic activity of GrB-(R201K)-TTC and the corresponding control construct GrB(R201K)-Mock was confirmed using the synthetic substrate Ac-IETD-*p*NA. The non-cleaved EGrB(R201K)-TTC protein showed no activity as expected (Fig 4C). An XTT-based cell viability assay using the mouse TTC-reactive hybridoma cell line 5E4 confirmed that the GrB(R201K)-TTC and TTC-MAPTau proteins showed no cross-reactivity with the murine cells and therefore no cytotoxicity. In contrast, the TTC-ETA' immunotoxin showed dose-dependent cytotoxicity towards these murine cells [26].

**Fig 5:**
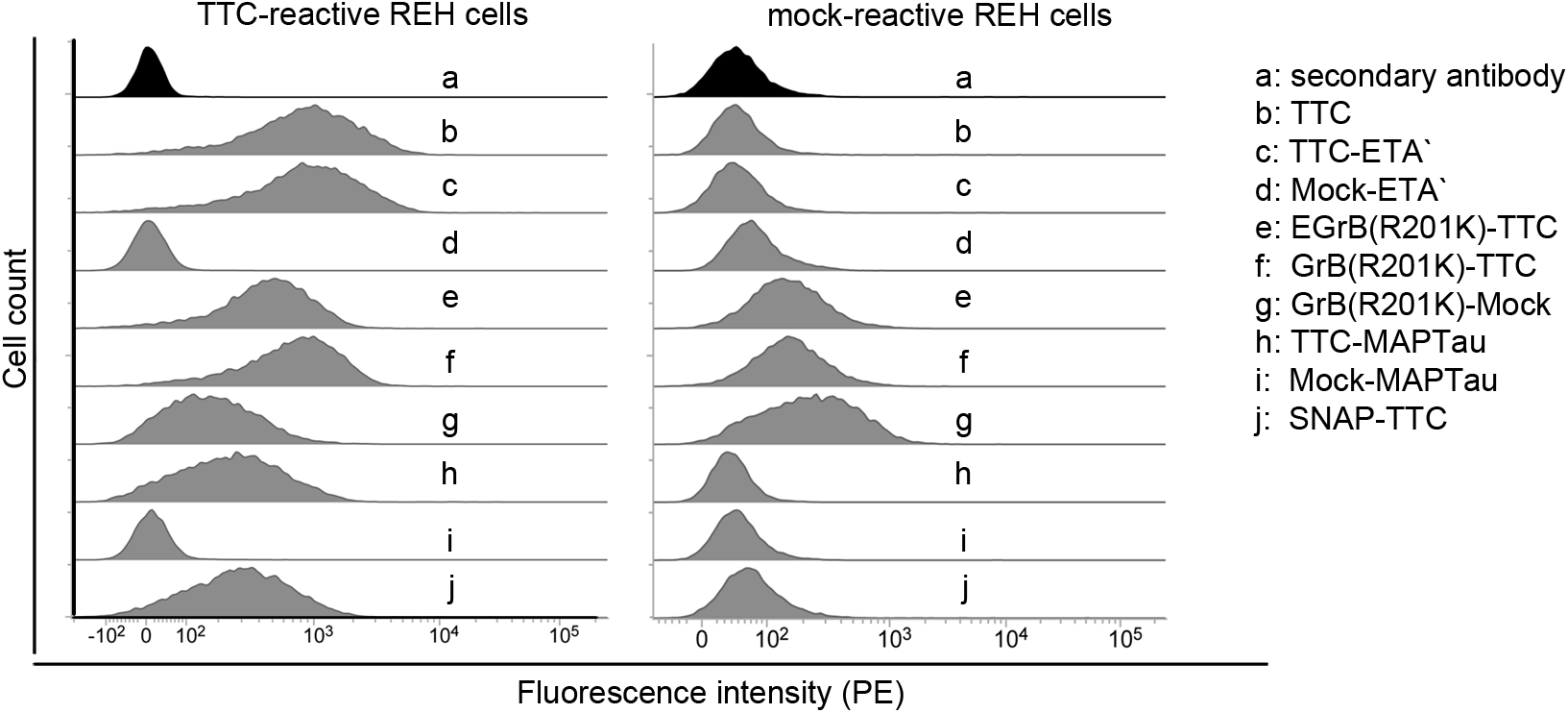
Binding of the TTC-based fusion proteins to TTC-reactive human lymphocytic REH cells. Equimolar amounts (100 nM) of the TTC-based fusion proteins and the control proteins (Mock-ETA', GrB(R201K)-Mock, and Mock-MAPTau) were analyzed by FACS. Proteins were detected using an anti-polyhistidine antibody conjugated to PE (diluted 1:100). The shift in the fluorescence signal from the background control (secondary antibody) indicated that the TTC-based fusion proteins bound to the TTC-reactive REH cells. There was no evidence of nonspecific binding to control REH cells.

The TTC-reactive REH cells were then used as a test system in order to characterize the novel TTC-based fusion proteins GrB(R201K)-TTC and TTC-MAPtau. Specific binding of the TTC-based fusion proteins to the TTC-reactive REH cells was compared to the mock-transfected REH control cells by FACS (Fig 5). Parallel experiments using mock-proteins with the corresponding effector domains confirmed that the specific binding was not affected by the effector domain of the fusion proteins. The mock-proteins, which have the same effector domain but a different cell-binding domain, showed no evidence of nonspecific binding to the transfected REH cells (Fig 5).

### Specific cytotoxicity and pro-apoptotic activity

**Fig 6:**
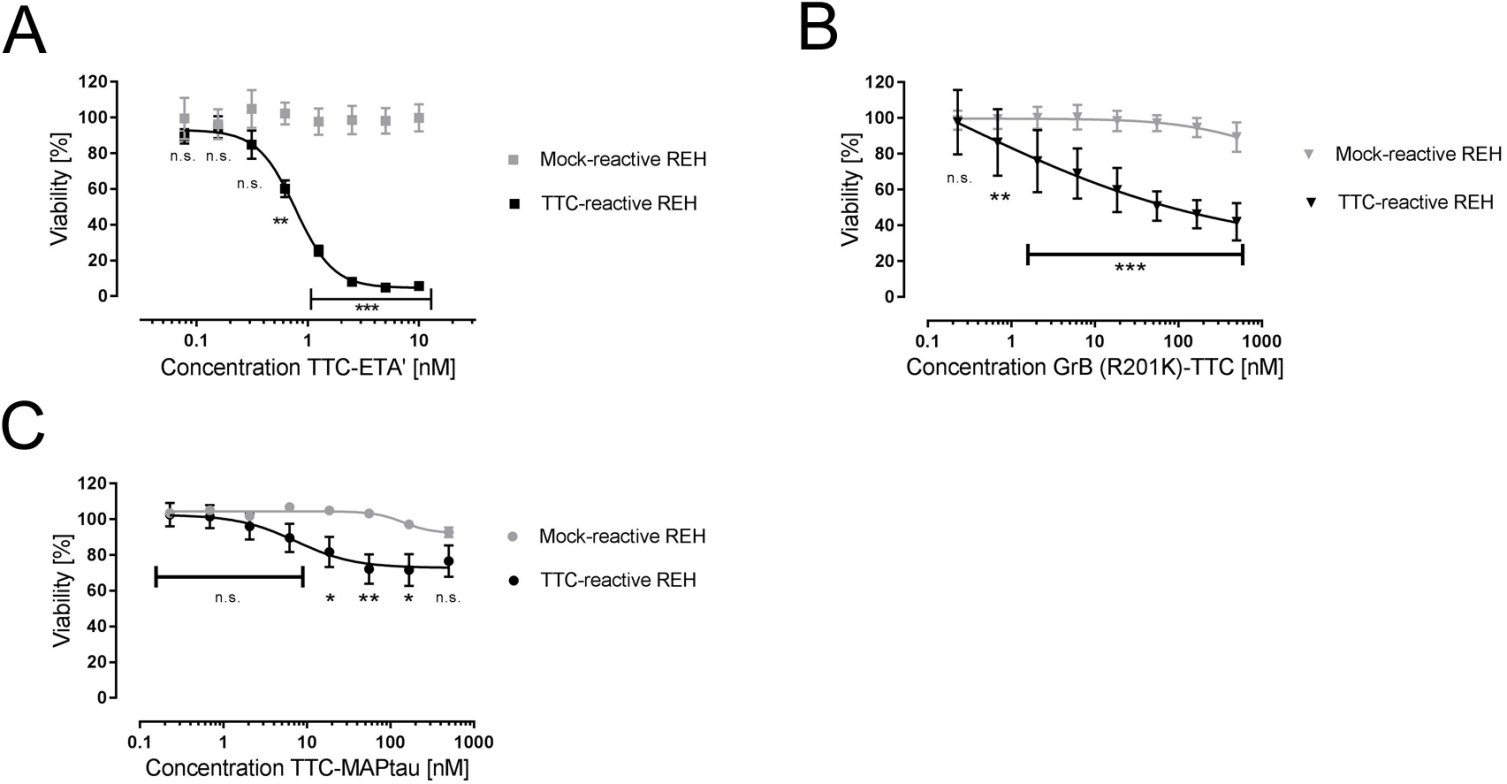
XTT cell viability assay to determine the cytotoxicity of TTC-based proteins. TTC-reactive REH cells (black) as well as the mock-transfected control REH cells (gray) were used to demonstrate the cytotoxicity of TTC-ETA' (**A**, ■), GrB(R201K)-TTC (**B**, ▼) and TTC-MAPTau (**C**, ●). The cells were incubated with an increasing concentration of the recombinant fusion proteins for 72 h at 37°C and 5% CO_2_ followed by an XTT cell viability assay. The EC_50_ value relative to untreated control cells (100% cell viability) and the positive control zeocin (0% cell viability) was calculated using the three-parameter dose-response curve fit equation with GraphPad Prism v5 software. The data are means ± SD of three independent experiments performed in triplicate (n = 3). Statistical significance was calculated by two-way ANOVA followed by Bonferroni’s post-hoc test (*** p < 0.001, ** p < 0.01, * p < 0.05, n.s.–not significant).

The XTT cell viability assay revealed that both the TTC-ETA' protein and the cytolytic GrB(R201K)-TTC protein showed dose-dependent toxicity towards REH cells after incubation for 72 h. As summarized in Table 2, the EC_50_ values were 0.76 ± 0.13 nM ($\bar{x}$ ± SD, n = 3) for TTC-ETA' and 5.0 ± 1.5 nM ($\bar{x}$ ± SD, n = 3) for GrB(R201K)-TTC, compared to untreated cells (100% cell viability) and the positive control zeocin (0% viability). A statistically significant reduction in cell viability was observed for both TTC-ETA' (p < 0.001, Fig 6A) and GrB(R201K)-TTC (p< 0.001, Fig 6B) compared to the mock-reactive REH control cells, analyzed by two-way ANOVA followed by Bonferoni’s post-hoc test. For TTC-MAPTau, concentrations above 18 nM showed a specific killing effect on the TTC-reactive REH cells, only at the highest applied concentration, the construct had a marginal impact on cell viability on mock-transfected REH cells (Fig 6C). The control TTC protein (without an effector domain) did not affect the viability of the TTC-reactive REH or control cells (S1 Fig).

**Table 2:** EC_50_ values of TTC-fusion proteins on TTC-reactive REH cells.

	EC_50_ ($\bar{x}$ ± SD)
**TTC-ETA`**	0.76 ± 0.13 nM
**GrB(R201K)-TTC**	5.0 ± 1.5 nM
**TTC-MAPTau**	not detectable

After the XTT cell viability assay, see Fig 6, the EC_50_ values of the different TTC-fusion proteins were calculated. They indicated the required protein concentration to achieve a 50% reduction of the cell viability.

**Fig 7:**
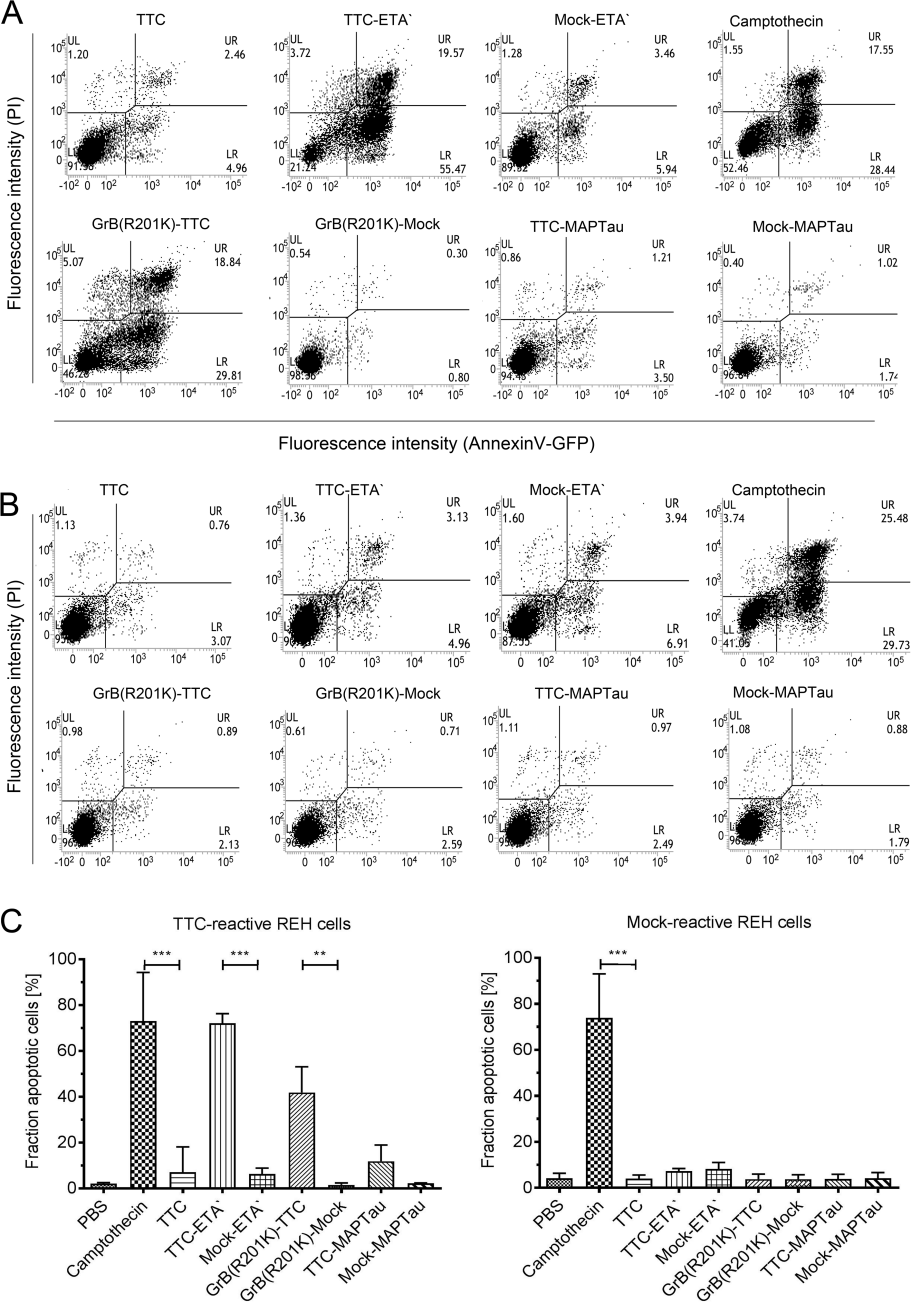
Annexin V/PI-staining of TTC-reactive REH cells and control cells treated with TTC fusion proteins and equivalent control proteins. The assay was carried out after incubating the TTC-reactive REH cells (A) or control REH cells (B) with 50 nM of each protein for 72 h at 37°C. The induction of apoptosis was measured by FACS after annexin V/PI staining. Representative dot blots are shown with the cells distributed as follows: lower left = viable cells, lower right = early apoptotic cells, upper right = late apoptotic cells. (C)The Induction of apoptosis following treatment with TTC-ETA' and GrB(R201K)-TTC. TTC-reactive REH cells (left) and the control REH cells (right) were incubated with 50 nM TTC-ETA', Mock-ETA', GrB(R201K)-TTC or GrB(R201K)-Mock, with a buffer-only negative control and a camptothecin positive control for 72 h at 37°C. The sum of early and late apoptotic cells ($\bar{x}$ ± SD) from three independent experiments carried out in duplicate is presented for each protein. The statistical significance was determined by one-way ANOVA followed by Bonferroni’s post-hoc test (***: p ≤ 0.001, **: p ≤ 0.01).

The ability of the TTC fusion proteins to induce apoptosis was measured by annexin V/PI staining. Briefly, the cells were incubated for 72 h at 37°C using 50 nM TTC, TTC-ETA', GrB(R201K)-TTC or TTC-MAPTau, or the corresponding mock-control proteins (Mock-ETA', GrB(R201K)-Mock, or Mock-MAPTau). Fig 7A shows representative dot blots for each protein tested against TTC-reactive-REH or control REH cells. The proportion of the TTC-reactive REH cell population induced to undergo apoptosis was ~72% for TTC-ETA' and ~45.5% for GrB(R201K)-TTC but only 20% for TTC-MAPTau (Fig 7B, left diagram). As expected, none of the proteins had a significant impact on the proportion of apoptotic cells in the control cell line (Fig 7B, right diagram).

## Discussion

Current strategies for the treatment of autoimmune diseases using monoclonal antibodies that target B cells show systemic effects. The targeting of CD19, CD20 or CD22 eliminates entire B cell subpopulations resulting in severe side effects such as a higher risk of concomitant infections [17, 19]. In contrast, our strategy targets the unique BCRs found on B cells. Antigen-based fusion proteins using ETA' or diphtheria toxin A (DTA) as cytotoxic domains have shown promising therapeutic effects against autoimmune diseases. For example, multiple sclerosis has been treated by targeting the autoantigen myelin oligodendrocyte glycoprotein (MOG) with immunotoxins containing ETA' or DTA, resulting in the specific depletion of the autoreactive cell population in mouse models [57, 58]. In our additional proof of concept study [26], we confirmed the specific targeting and depletion of human memory B cells using an antigen-specific fusion protein containing ETA'.

Immunotoxins containing bacterial or plant toxins can potentially trigger an immune response in humans, which limits the therapeutic application to one or two doses before the patient eventually develops neutralizing antibodies against effector domain or the cell-binding domain [29, 59]. To overcome this challenge, a new generation of immunotoxins has been developed containing human pro-apoptotic proteins such as granzyme B, angiogenin or MAPTau [21, 27–30, 54, 60, 61]. We therefore used the established pMS vector system [53] to prepare fusion constructs combining two human effector proteins with TTC as a model antigen, resulting in two novel TTC-based fusion proteins: EGrB(R201K)-TTC and TTC-MAPTau. These proteins were expressed successfully following the transfection of HEK 293T cells, and the expression yield of the protein EGrB(R201K)-TTC we achieved (4.5 mg/l) is comparable to the yields of other granzyme B fusion proteins produced in HEK 293T cells, ranging from 1 to 30 mg/l [30, 32, 62]. The yield of TTC-MAPTau was 1.6 mg/l, which is comparable to other MAPTau fusion proteins such as Ki4scFv-MAP and anti-EpCAMscFv-MAP produced in *E*. *coli* with yields of up to 1 mg/l [60, 63, 64]. Eukaryotic expression systems offer the advantage of endotoxin-free fusion protein production, so that additional purification steps are unnecessary. We confirmed the enzymatic activity of the granzyme B fusion protein using an appropriate substrate assay (Fig 4C). However, neither EGrB(R201K)-TTC nor TTC-MAPTau were cytotoxic towards the murine TTC-reactive hybridoma cell line 5E4, due to their species-dependent activity (Fig 4D). A novel TTC-reactive human lymphocytic cell line was therefore necessary for the *in vitro* characterization of the cytolytic fusion proteins.

Stable mammalian cell lines presenting or expressing specific recombinant proteins can be produced using diverse techniques such as retroviral/lentiviral vectors [65], transient transfection with plasmid DNA [66] or transposon-based gene transfer systems [44–47, 67]. Several reports have demonstrated the generation of tailor-made human lymphocytic cells lines using transposon-based gene transfer [39, 44, 47, 68–70]. We used Transpo-mAb^TM^ technology to establish a human cell line displaying a TTC-reactive BCR on the cell surface. We found that it was possible to transfect mammalian lymphocytic REH cells (precursor B cells) and achieve the same transfection efficiency usually reported for electroporation [39]. This allowed us to express a full-length antibody sequence that underwent all the correct folding and post-translational modifications required for the presentation of a functional antibody on a human B cell line. Transposon-based gene transfer provides a rapid and straightforward method to generate a human artificial cellular test system featuring any antibody of interest requiring only 2–3 months of laboratory work. The generation of a B cell line presenting a human antibody would be much more expensive and laborious using hybridoma technology [71].

The REH cells maintained their lymphocytic behavior after transfection, as shown by the rapid receptor-mediated internalization of the SNAP-TTC-BG-647 protein bound to the TTC-reactive BCR (Fig 3). The efficiency of internalization was comparable to the rate of BCR endocytosis observed in isolated lymphocytic cells [72–74]. The novel TTC-reactive REH cell line continued to present the specific anti-TTC antibody on the cell surface for a cultivation period of 1–2 months, which is comparable to previous reports [39]. The novel human TTC-reactive lymphocytic REH cell population can thus serve as valuable *in vitro* test system for the characterization of novel cytolytic fusion proteins such as GrB(R201K)-TTC and TTC-MAPTau. Transpo-mAb^TM^ technology therefore appears to be ideal for the high-throughput analysis of novel therapeutic antibodies and fusion proteins, and also for epitope mapping analysis, not only in the field of autoimmune disorders (characterized by a low frequency of autoreactive memory B cells) but also for the characterization of specific memory B cells in the field of infectious diseases such as malaria, Ebola and dengue fever.

We used the new artificial cellular test system for the *in vitro* characterization of the TTC-based fusion proteins starting with their cell-binding properties. As expected, all TTC-based proteins bound specifically to the BCR of the TTC-reactive REH cells but not to the mock-transfected control REH cells (Fig 5). A small amount of granzyme B binds nonspecifically to the REH cells due to the high isoelectric point of the protein and the positive surface charge of the cells, as indicated by a small shift in fluorescence signal [75–77]. However, the GrB(R201K)-TTC fusion protein predominantly bound specifically to the TTC-reactive REH cells.

The TTC-ETA' protein was used as a positive control to show the specific binding and depletion of the human TTC-reactive REH cells (Figs 5 and 6A) with a low EC_50_ value in the picomolar range. In contrast, the EC_50_ value of GrB(R201K)-TTC against REH cells was 5 nM (Fig 6B), which is in the same range or slightly lower than other granzyme B fusion proteins targeting cancer cells such as GbR201K-Ki4(scFv) against L540cy cells (EC_50_ = 1.7 nM), GbR201K-scFv1711 against RD cells (EC_50_ = 21.1 nM), or the anti-malarial activity of granzyme B (EC_50_ = 176 nM) [30, 32, 54, 56, 78].

The TTC-MAPTau protein was much less potent than GrB(R201K)-TTC and reduced cell proliferation by only 20% (Fig 6C). This may reflect the low proliferation rate of the transfected REH cells: under these circumstances, the interruption of dynamic microtubule assembly and disassembly by MAPTau might not induce apoptosis within the time frame of the experiment. Cells with high proliferation rates, such as cancer cells, are more sensitive to MAPTau, as demonstrated in earlier studies testing MAPTau fusion proteins as candidates for cancer therapy. For example, EGF-MAP achieved a half-maximal inhibitory concentration (IC_50_) of 1 μM against pancreatic L3.6pI cells, and Ki(scFv)-MAP achieved an IC_50_ value of 53 nM against the Hodgkin’s lymphoma cell line L540cy [60, 63, 64]. Autoreactive memory B cells are also characterized by slow proliferation, so MAPTau fusion proteins may be less useful than granzyme B fusion proteins in this context. However, if the memory B cells contact the autoantigen and differentiate into rapidly-proliferating plasma blasts [79], the MAPTau protein effector domain could have a more potent effect on the dynamic behavior of microtubules resulting in the rapid induction of apoptosis.

GrB(R201K)-TTC induced apoptosis much more efficiently than TTC-MAPTau in human lymphocytic REH cells (Fig 7). We have therefore demonstrated that human artificial antigen-specific B lymphocytes can be targeted and eliminated using a TTC-based fusion protein with GrB(R201K) as the effector domain. This suggests that such antigen-based fusion proteins could also be suitable for the antigen-specific elimination of autoreactive human B cells *in vivo* to as a novel strategy for the treatment of autoimmune disorders.

## Conclusion

Transpo-mAb^TM^ technology facilitates the simple and rapid generation of antigen-specific BCRs on the surface of mammalian cells using *piggyBac* transposase. We expressed a chimeric TTC-specific antibody on the surface of human lymphocytic REH cells, in which the constant regions were human in origin and only the variable regions (V_L_/V_H_) were derived from the murine antibody. The human lymphocytic REH cells provide a valuable *in vitro* test system to characterize antigen-specific human cytolytic fusion proteins. This technology has the potential to facilitate high-throughput screening for novel antibodies, antibody–drug conjugates and cytolytic fusion proteins applicable to a wide range of diseases, including cancer, infections and autoimmune diseases.

Our antigen-specific fusion protein containing the granzyme B (R201K) mutant as the toxic component efficiently eliminated human B cells as demonstrated by annexin V/PI staining and XTT-based cell proliferation assays. These results confirm that the granzyme B (R201K) mutant is a promising effector domain for the treatment of B cell-driven autoimmune diseases such as SLE, lupus nephritis and multiple sclerosis. The potential of the GrB(R201K)-TTC fusion protein for the elimination of autoreactive human B cells must now be tested *in vivo*.

## Supporting information

S1 Fig XTT cell viability assay to determine the cytotoxicity of TTC protein. TTC-reactive REH cells (black) as well as the mock-transfected control REH cells (gray) were used to demonstrate the cytotoxicity of TTC protein without a fused effector domain (♦).The cells were incubated with an increasing concentration of the recombinant fusion proteins for 72 h at 37°C and 5% CO_2_ followed by an XTT cell viability assay. As no cytotoxicity could be measured using the applied concentrations, no EC_50_ value could be determined.
(TIF)

S1 Table Raw data of the granzyme B substrate assay. (XLSX)

S2 Table Raw data of the XTT assay. (XLSX)

S3 Table Raw data of the annexin V assay. (XLSX)
