# Supplementary figures and images for: Generation of an artificial human B cell line test system using Transpo-mAbTM technology to evaluate the therapeutic efficacy of novel antigen-specific fusion proteins
Source: PLoS One. 2017 Jul 13;12(7):e0180305. doi: 10.1371/journal.pone.0180305 (PMC5509223; doi:10.1371/journal.pone.0180305)

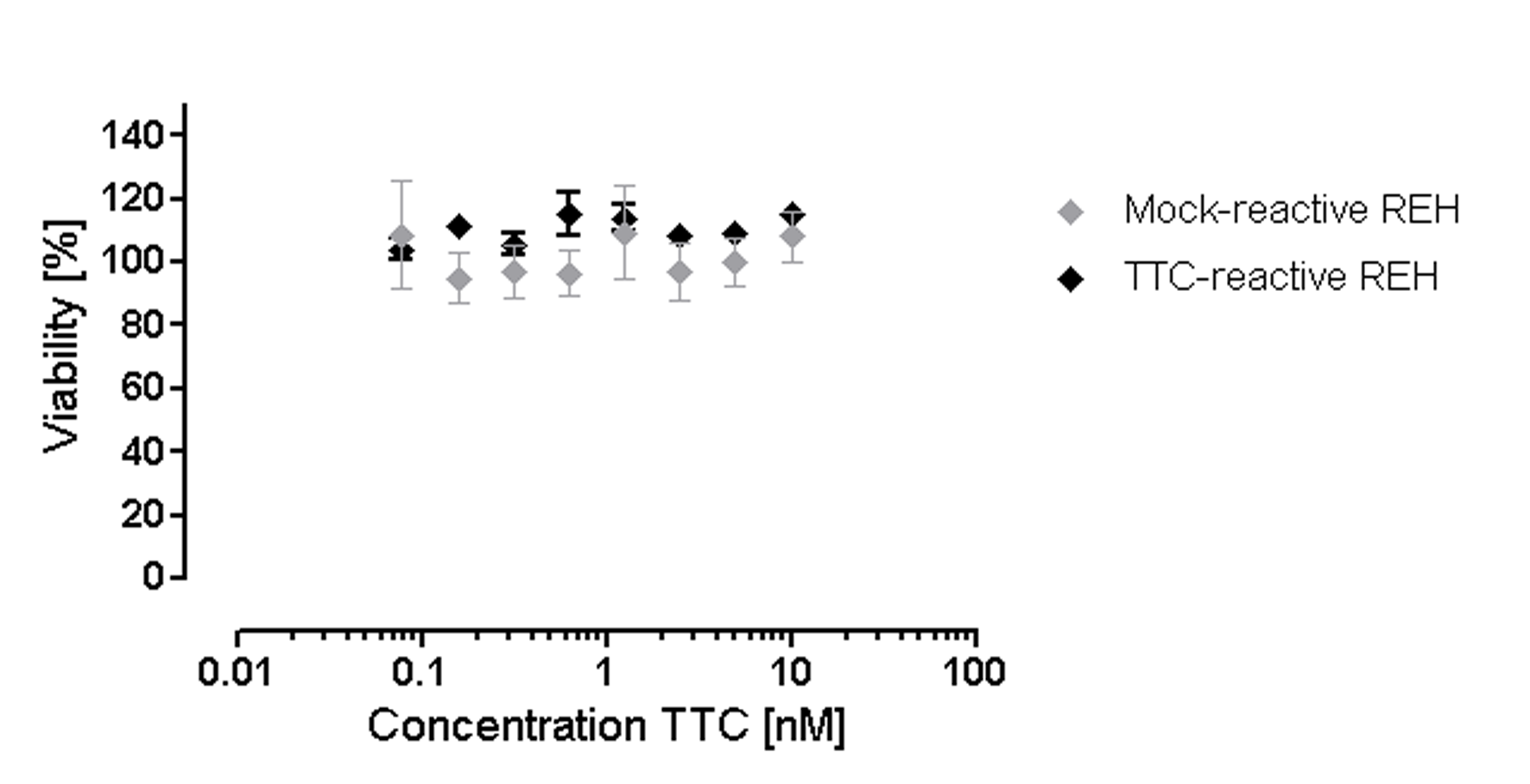

Supplement: S1 Fig — TTC-reactive REH cells (black) as well as the mock-transfected control REH cells (gray) were used to demonstrate the cytotoxicity of TTC protein without a fused effector domain (♦).The cells were incubated with an increasing concentration of the recombinant fusion proteins for 72 h at 37°C and 5% CO2 followed by an XTT cell viability assay. As no cytotoxicity could be measured using the applied concentrations, no EC50 value could be determined. (TIF) [file pone.0180305.s001.tif]
